# Tmem39b promotes tumor progression and sorafenib resistance by inhibiting ferroptosis in hepatocellular carcinoma

**DOI:** 10.32604/or.2024.046170

**Published:** 2024-07-17

**Authors:** MING ZHUANG, XUE ZHANG, LU LI, LIMING WEN, JIAMIN QIN

**Affiliations:** Department of Gastroenterology, Sichuan Mianyang 404 Hospital, Mianyang, 621000, China

**Keywords:** TMEM39b, Sorafenib, Ferroptosis, Hepatocellular carcinoma

## Abstract

Hepatocellular carcinoma (HCC) poses a significant threat to human health. Resistance to sorafenib in the chemotherapy of HCC is a common and significant issue that profoundly impacts clinical treatment. While several members of the transmembrane (TMEM) protein family have been implicated in the occurrence and progression of HCC, the association between TMEM39b and HCC remains unexplored. This study revealed a significant overexpression of TMEM39b in HCC, which correlated with a poor prognosis. Subsequent investigation revealed that RAS-selective lethal 3 (RSL3) induced pronounced ferroptosis in HCC, and knocking down the expression of TMEM39b significantly decreased its severity. Similarly, following the induction of ferroptosis in HCC by sorafenib, knocking down the expression of TMEM39b also decreased the severity of ferroptosis, enhancing HCC tolerance to sorafenib. In conclusion, we propose that TMEM39b promotes tumor progression and resistance to sorafenib by inhibiting ferroptosis in HCC.

## Introduction

In 2020, the International Agency for Research on Cancer reported that HCC had become the sixth most frequently diagnosed cancer and the third leading cause of cancer-related mortality globally, with approximately 906,000 new cases and 830,000 deaths [1]. China’s National Clinical Research Center for Cancer recently published epidemiological data on liver cancer in China. The report indicated approximately 389,000 cases of liver cancer and 336,000 associated deaths in China in 2016, with significantly higher morbidity and mortality among male patients compared to female patients [2]. In the early stage of liver cancer, specific clinical symptoms are often absent, leading to missed opportunities for early surgical intervention upon diagnosis [3,4]. Sorafenib, approved by the U.S. Food and Drug Administration (FDA) in 2007, was the first oral multikinase inhibitor for treating advanced HCC and remains the most commonly prescribed first-line chemotherapy for advanced liver cancer patients [5,6]. However, in clinical practice, many patients receiving sorafenib have quickly developed resistance [7]. Numerous studies have explored sorafenib resistance, yet current research has not provided a definitive solution to this Issue [7]. Recent studies have revealed that sorafenib can inhibit cystine glutamate transporter receptor-induced ferroptosis in HCC [8,9]. Dixon et al. [10] first proposed the concept of cellular ferroptosis, which mainly involves Fe^2+^ or ester oxygenase catalyzing the lipid peroxidation of unsaturated fatty acids on the cell membrane, leading to non-apoptotic cell death characterized by the accumulation of reactive oxygen species (ROS) in the cell [11]. Research has shown that the toxic effect of sorafenib on HCC cells can be mitigated by depleting intracellular iron reserves using the iron-chelating agent deferoxamine [12]. Sorafenib, as a multi-target kinase inhibitor, can induce apoptosis in various cancer cells and inhibit angiogenesis [13]. It is the sole drug among all current kinase inhibitors capable of inducing cell ferroptosis to eradicate tumor cells [9]. TMEM proteins are a broad category of proteins capable of binding to cell membranes or organelles [14]. Increasing evidence supports the crucial role of TMEM family proteins in the onset and progression of cancer [15]. TMEM147 has been identified as a potential biological marker for poor prognosis in HCC [16]. Duan et al. found that TMEM106C was overexpressed in liver cancer, and inhibition of TMEM106C significantly inhibited the proliferation and metastasis of liver cancer by targeting centromere protein M and deleted in liver cancer-1 [17]. Rao reported that TMEM205 could potentially enhance the prognosis of patients with HCC by decreasing the levels of immunosuppressive cells and facilitating the infiltration of cytotoxic T cells into the tumor microenvironment [7]. The most recent study also revealed a strong association between TMEM65 and ferroptosis in various common tumors [18]. Through Gene Expression Omnibus datasets, we found that the expression of TMEM39b was significantly different in liver cancer tissues and normal tissues. Nevertheless, there is currently no evident literature indicating the mechanism through which TMEM39b affects the prognosis of HCC.

## Methods and Materials

### The gene expression omnibus (GEO) database was used for data verification

Four GEO datasets (GSE36776, GSE60502, GSE62232, and GSE36776) were selected to assess the transcript levels of TMEM39b in both cancer and adjacent tissues of HCC patients.

### Analysis of TMEM39b gene expression

The Tumor Immune Estimation Resource (TIMER) database (https://cistrome.shinyapps.io/timer/) and TNMplot database (https://tnmplot.com/analysis/) were utilized to analyze TMEM39b expression in various cancers, including HCC.

### Kaplan-Meier survival analysis

The median expression of the TIMER gene was used to stratify the patient data into high and low TMEM39b expression groups. The Kaplan-Meier survival curves (http://kmplot.com/analysis/) were employed to assess the impact of TMEM39b expression levels on the survival of HCC patients.

### Cell culture

The human HCC cell line, HepG2, was obtained from the China Center for Type Culture Collection (Wuhan, China), and Huh7 was from the American Type Culture Collection (Manassas, VA, USA). Miha was from China Center for Type Culture Collection (Wuhan, China). The cells were cultured in Dulbecco’s modified eagle medium (high-glucose) containing 10% fetal bovine serum (Gibco, Australia Origin) and 1% penicillin-streptomycin (Gibco, New York, USA). All cells were maintained at 37°C and 5% CO_2_. Configure RSL3 (Abcam, Cambridge, UK) as a solution with a concentration of 3 µM according to the manufacturer’s instructions. After attachment overnight, HCC cells were washed twice with PBS and the medium was replaced by medium with 3 µM RSL3 for 24 h.

### Cell viability assay

HuepG2 cells (1 × 10^4^ cells per well) and Huh7 cells (2 × 10^4^ cells per well) were seeded in 96-well plates. After culturing for 24 h, the cells were intervened at the specified time point. The Cell Counting Kit-8 (CCK-8) (Beyotime, Shanghai, China) was used to measure cell survival, while the Click-iT™ EdU Imaging Kit (Thermo Fisher Scientific, Massachusetts, USA) was utilized to assess the proliferative capacity of tumor cells, following the manufacturer’s instructions.

### Colony formation

After the intervention, cells were arranged into various experimental designs and then seeded at a density of 800 cells per well in 6-well plates. The medium was replaced every 3 days, and the culture was maintained until the number of most dominant single clone cells exceeded 50%. Subsequently, the cells were rinsed with phosphate-buffered saline (PBS), treated with 1 mL of 4% paraformaldehyde for 30 min, and then washed again with PBS. Following this, 1 mL of crystal violet was added to each well and the cells were stained for 20 min.

### Cell scratch assay

HepG2 and Huh7 cells were seeded in 6-well plates. Once the cell confluence reached 90%, scratches were made using a 200 μL sterilized pipette tip. The cells were intervened at a specific time point. Images of the same scratch area at 0 and 24 h were captured using an inverted microscope.

### Transwell detection

The cells were starved for 12 h, then treated with trypsin, and subsequently washed twice with PBS before re-suspension. A total of 2.5 × 10^5^ cells were seeded into the upper chamber, and drugs were added according to the experimental group requirements. The lower chamber was added with Dulbecco’s modified eagle medium containing 20% fetal bovine serum and 1% penicillin-streptomycin. Following incubation at 37°C for 24 h, the Transwell plates were rinsed with PBS, fixed with formaldehyde, and stained with crystal violet. Uninvaded cells were removed using cotton swabs. Finally, the cells were observed and imaged under an inverted microscope.

### Detection of intracellular Fe^2+^, ROS and 4-HNE level

HepG2 and Huh7 cells were seeded into 6-well plates at a density of 4 × 10^5^ cells per well, followed by drug intervention for 24 h. Intracellular Fe^2+^ levels were measured using FerroOrange (Goryo Chemical Inc, Hokkaido, Japan) following the manufacturer’s protocol. Additionally, intracellular ROS levels were assessed using the DHE (Dihydroethidium) Assay Kit-ROS, and oxidative stress biomarker (4-HNE) detection were assessed using the Lipid Peroxidation Assay (Abcam, Cambridge, UK), following the respective manufacturer’s protocols.

### Western blot

The cells were lysed and homogenized using a RIPA lysis buffer containing protease inhibitors (Thermo Fisher Scientific, Massachusetts, USA). The protein concentration was then measured with the Pierce BCA Protein Assay Kit (Thermo Fisher Scientific, Massachusetts, USA) and denatured at 99°C for 5 min. The proteins were separated by sodium dodecyl sulfate-polyacrylamide gel electrophoresis and then transferred to a polyvinylidene fluoride (PVDF) membrane. The PVDF membranes were then blocked using 5% skim milk for 1 h. The PVDF membrane was then incubated with the primary antibodies against TMEM39b (Thermo Fisher Scientific, Massachusetts, USA) and GAPDH (Abcam, Cambridge, United Kingdom) at 4°C overnight, followed by blocking with the secondary antibody (Abcam, Cambridge, UK) at room temperature for 1 h. After washing with tris buffered saline tween, the bands were detected using Chemiluminescent Horse Radish Peroxidase Substrate (Merck KGaA, Darmstadt, Germany), and the intensity of each protein band was calculated using Image J for bio-visualization.

### Immunohistochemistry

We obtained liver cancer tissues and adjacent tissues from patients who had undergone liver cancer resection in our hospital. Informed consent was obtained from these patients. Paraffin-embedded specimens were deparaffinized at 56°C for 2 h and rehydrated using a gradient of ethanol, followed by antigen retrieval in a pressure cooker with citrate buffer (pH 6.0). Endogenous peroxidase activity was inactivated by pretreating with 3% H_2_O_2_ for 25 min at 25°C. The sections were then incubated with primary antibodies against TMEM39b (Thermo Fisher Scientific, Massachusetts, USA) overnight at 4°C and secondary antibodies (Thermo Fisher Scientific, Massachusetts, USA) for 60 min at 25°C. Finally, the sections were stained with diaminobenzidine (Thermo Fisher Scientific, Massachusetts, USA) and counterstained with hematoxylin (Beyotime, Shanghai, China).

### Immunofluorescence

HepG2 cells were plated at a density of 1 × 10^5^ cells per well in a 24-well plate. After drug intervention at 37°C for 24 h, the cells were washed three times with PBS and then fixed with 4% paraformaldehyde for 10 min. After washing twice with PBS, the cells were permeabilized with 0.5% Triton X-100 (Merck KGaA, Darmstadt, Germany) on ice for 8 min. Following washing three times with PBS, the cells were blocked with 5% goat serum for 2 h at room temperature and then incubated with primary antibodies against GPX4 (Thermo Fisher Scientific, Massachusetts, USA) overnight at 4°C. The cells were washed with PBS three times before being incubated with a secondary antibody (Thermo Fisher Scientific, Massachusetts, U.S) at room temperature for 1 h. Then, they were washed again with PBS three times, and a mounting medium was added to mount the coverslips on glass slides. Immunofluorescence staining was visualized under a fluorescence microscope. The experiment was repeated with Huh7 cells at a density of 2 × 10^5^ cells per well.

### RNA isolation and real-time quantitative PCR (qPCR)

Total RNA was extracted from cells using a TRIZOL Regent Kit (Takara, Tokyo, Japan) according to the manufacturer’s protocol. Complementary DNA was synthesized using a standard reverse transcription kit (Takara, Tokyo, Japan) according to the manufacturer’s protocol. qPCR was performed using a QuantStudio5 system (Thermo Fisher Scientific, Massachusetts, USA) with SYBR Green PCR kit (Toyobo Life Science, Osaka, Japan). The thermo‑cycling conditions were as follows: 95°C for 30 s, 95°C for 5 s and 60°C for 30 s, for 40 cycles. Results were analyzed using the 2^−ΔΔCq^ method. GAPDH was regarded as the control. Primer sequences were as follows: TMEM39b forward, 5′-CAGTGCATCGGTGACCAGT-3′ and reverse, 5′-CAGTGCTGTAGAGGCACAAC-3′; GAPDH forward, 5′-GTTCAACGGCACAGTCAAG-3′ and reverse, 5′-TACTCAGCACCAGCATCAC-3′.

### Transfection and TMEM39b small RNA interference

siRNAs of TMEM39b and negative control siRNA were synthesized by Beyotime (Shanghai, China). The TMEM39b siRNA sequences were 5′-GCACACAAGACAGCTGTATGG-3′. The HCC cells were transfected with the siRNA of TMEM39b in subsequent experiments. The transfection experiments used the FUGENE® HD (Roche, Basel, Switzerland) transfection reagent following the manufacturer’s protocol.

### Statistical analysis

The data were analyzed with *t*-tests and one-way or two-way ANOVA, and the results are expressed as mean values ± standard deviation (SD). A value of *p* < 0.05 was considered to be statistically significant.

### Ethical approval

All studies have been approved by the Ethics Committee of Sichuan Mianyang 404 Hospital (Approval Number: 23-013). Informed consent was obtained from all patients, following the Helsinki Protocol.

## Results

### TMEM39b expression across cancers and in HCC

**Figure 1:**
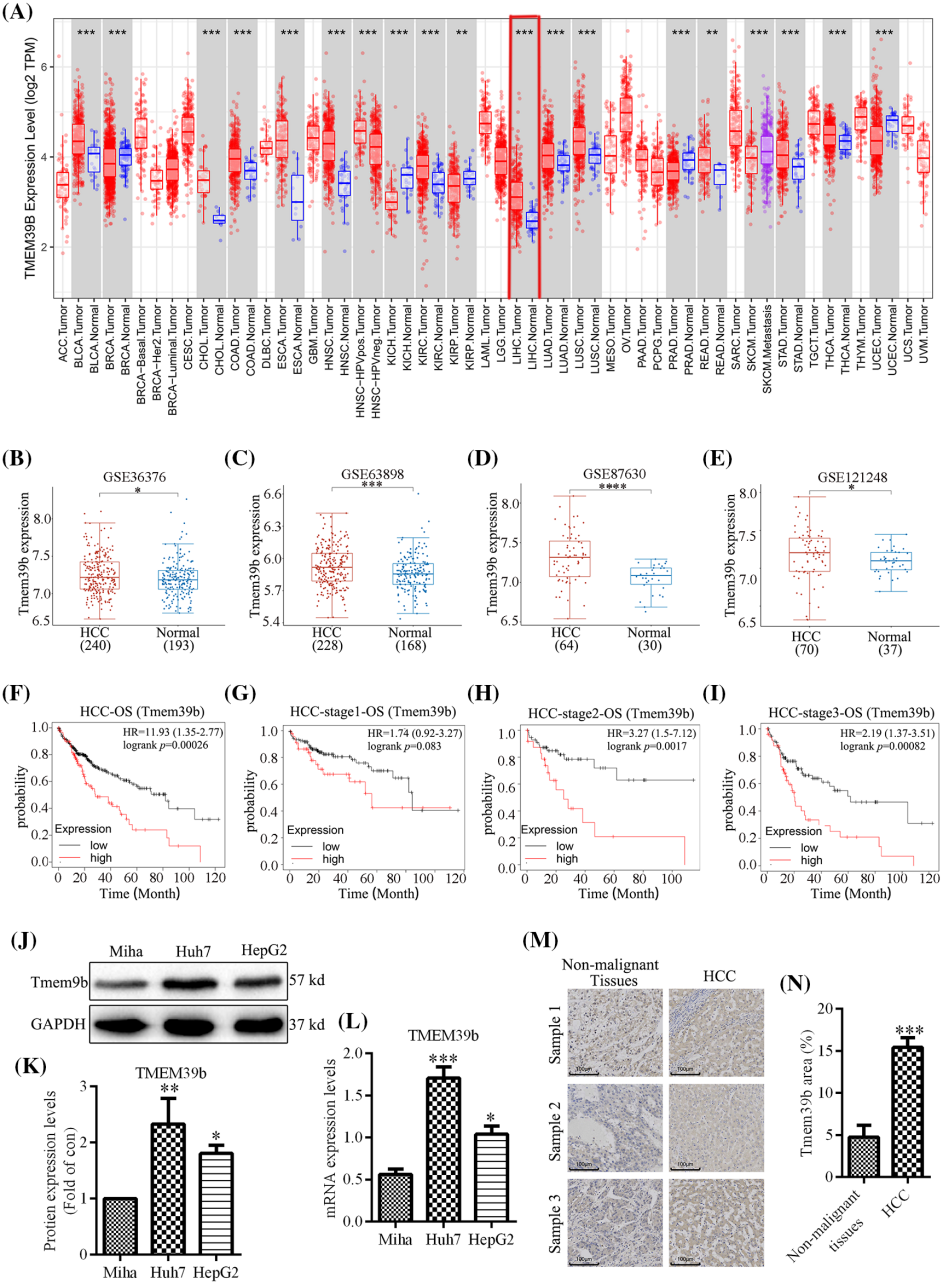
TMEM39b expression across cancers and in HCC. (A) The expression of TMEM39b in various cancer tissues and normal tissues. (B–E) The expression difference of TMEM39b in HCC and normal liver tissues was identified by the GEO database. (F–I) The relationship between the expression level of TMEM39b and the prognosis of HCC. (J–N) The expression level of TMEM39b in HCC and normal liver tissues was verified. *p < 0.05, **p < 0.01, ***p < 0.001, ****p < 0.0001 *vs*. HCC or non-malignant tissues.

The Wilcoxon rank-sum test was employed to compare the expression of TMEM39b across different cancer types using TIMER and TNMplot data. As shown in Fig. 1A, TMEM39b expression was significantly elevated in these cancer types, including HCC, compared to their respective normal tissues. TMEM39b expression was evaluated in four distinct GEO datasets (GSE36776, GSE60502, GSE62232, and GSE36776), confirming its elevated levels in HCC (Figs. 1B–1E). Elevated TMEM39b expression was associated with poor prognosis of HCC across various stages (Figs. 1F–1I). The mRNA and protein expression levels of TMEM39b were assessed in Huh7, HepG2, and Miha. Results from qPCR, Western blot, and immunohistochemistry confirmed the elevated expression of TMEM39b in Huh7 and HepG2 (Figs. 1J–1N).

### TMEM39b enhances HCC cell proliferation and migration

**Figure 2:**
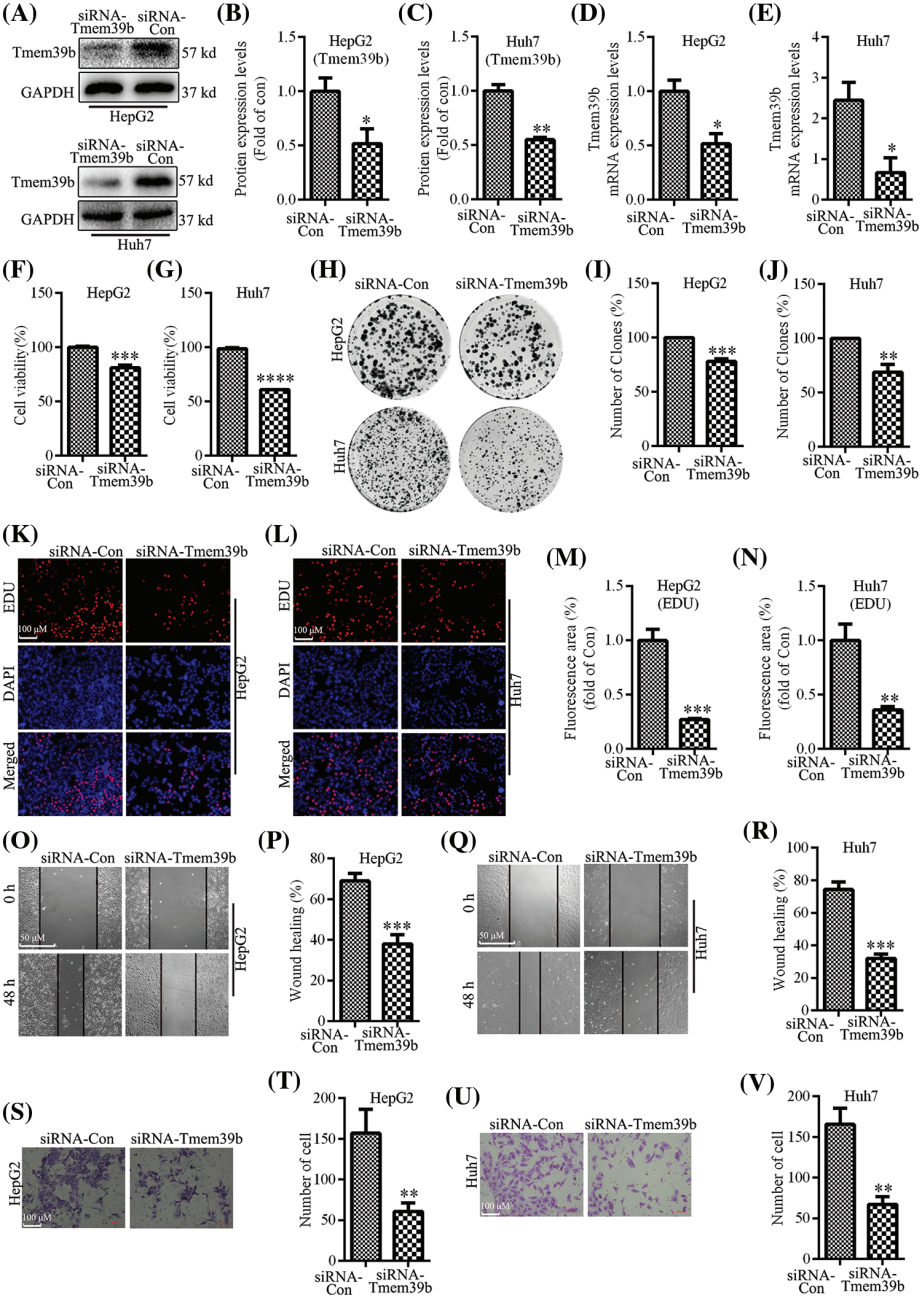
TMEM39b enhances HCC cell proliferation and migration. (A–E) The successful construction of HCC with low expression of TMEM39b was verified. (F–G) Comparison of the cell activity between the siRNA-Con group and the siRNA TMEM39b group. (H–N) Comparison of the proliferative activity between the siRNA-Con group and the siRNA TMEM39b group. (O–V) Comparison of the migration ability of HCC between the siRNA-Con group and the siRNA TMEM39b group. *p < 0.05, **p < 0.01, ***p < 0.001, ****p < 0.0001 *vs*. siRNA-Con.

We successfully constructed HepG2 and Huh7 cells with low TMEM39b expression by siRNA transfection. TMEM39b expression was verified by Western blot and real-time quantitative PCR (Figs. 2A–2E). The cell activity of HCC in the TMEM39b low expression group (si-TMEM39b group) was significantly lower than that in the control group (si-con group) (Figs. 2F and 2G). The colony formation assay was used to determine cell proliferation, revealing that the cell proliferation in the si-TMEM39b group was significantly lower than that of the si-con group (Figs. 2H–2J). Consistent results were obtained by EdU kit (Figs. 2K–2N). Transwell and scratch assays revealed that the migration rate of HCC in the si-TMEM39b group was significantly slower than that in the si-con group (Figs. 2O–2V). This suggests that TMEM39b significantly enhances the proliferation and migration of HCC.

### Correlation of TMEM39b expression with the ferroptosis-related gene expression in HCC

**Figure 3:**
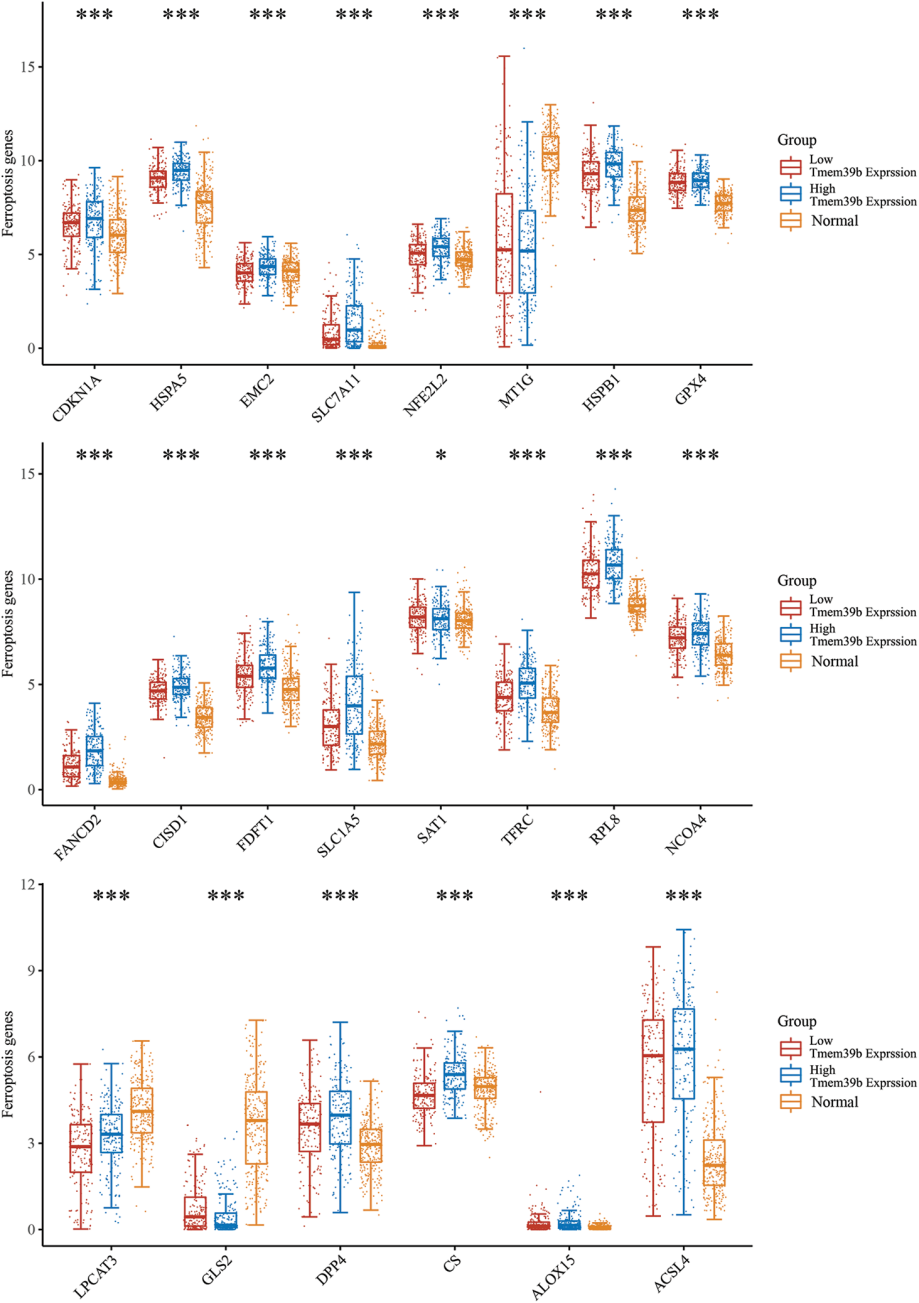
Correlation of TMEM39b expression with the ferroptosis-related gene expression in HCC through cancer genome atlas database screening. It was found that GPX4 expression was significantly correlated with TMEM39b. *p < 0.05, ***p < 0.001.

We screened the expression of ferroptosis-related genes in HCC by using the Cancer Genome Atlas database. We found that the expression of glutathione peroxidase 4 (GPX4) was significantly correlated with MEM39b (Fig. 3). A large number of studies have shown that GPX4 plays a key role in ferroptosis [19]. GPX4 is a target protein of RAS-selective lethal 3 (RSL3), which can inhibit GPX4 and promote the occurrence of ferroptosis. This suggests that TMEM39b may block the targeted effect of RSL3 on GPX4 by inhibiting RSL3, thereby inhibiting ferroptosis of HCC cells.

### si-TMEM39b promotes RSL3-induced ferroptosis in HCC cells

**Figure 4:**
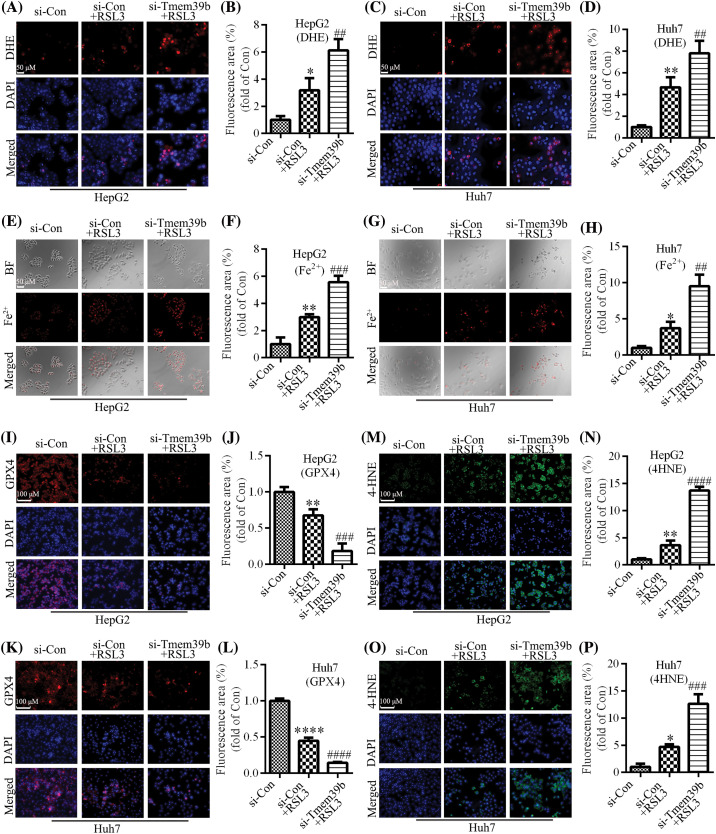
si-TMEM39b promotes RSL3-induced ferroptosis in HCC cell. (A–D) ROS levels in the si-Con group, si-Con + RSL3 group, and si-TMEM39b + RLS3 group. (E–H) Fe^2+^ content in the si-Con group, si-Con + RSL3 group, and si-TMEM39b + RLS3 group. (I–L) GPX4 content in the si-Con group, si-Con + RSL3 group, and si-TMEM39b + RLS3 group. (M–P) The 4-HNE content in the si-Con group, si-Con + RSL3 group, and si-TMEM39b + RLS3 group. ^#^*p* < 0.05, ^##^*p* < 0.01, ^###^*p* < 0.001, ^####^*p* < 0.0001 *vs*. Si-Con+RSL3. *p < 0.05, **p < 0.01, ***p < 0.001, ****p < 0.0001 *vs*. Si-Con.

RSL3 is well-established as an inducer of ferroptosis. As displayed in Figs. 4A–4D, RSL3 led to an increase in ROS in HCC cells. Interestingly, treatment of HCC with RSL3 based on TMEM39b knockdown resulted in a more pronounced increase in ROS in HCC cells. The Fe^2+^ content increased in HCC cells treated with RSL3, and the Fe^2+^ content of HCC cells increased more profoundly in the si-TMEM39b group (Figs. 4E–4H). The increase in Fe^2+^ content can promote cell ferroptosis. Additionally, TMEM39b may have an inhibitory effect on cell ferroptosis, as inhibition of its expression revealed an increase in Fe^2+^ content and a reduction in RSL3-induced GPX4 expression (Figs. 4I–4L), suggesting that inhibiting TMEM39b may promote RSL3-induced ferroptosis. 4-HNE, a by-product of lipid peroxidation, is commonly acknowledged as a reliable marker of oxidative stress. Our study revealed that RSL3 treatment induced an upregulation of 4-HNE expression in HCC cells. Furthermore, knocking down TMEM39b expression followed by RSL3 treatment led to a more pronounced increase in 4-HNE expression (Figs. 4M–4P). These results suggest that inhibiting the expression of TMEM39b is conducive to the induction of lipid peroxidation in HCC by RSL3. In addition, si-TMEM39b promotes RSL3-induced HCC ferroptosis, indicating that TMEN39b in ordinary HCC may inhibit RSL3-mediated HCC ferroptosis compared to low TMEM39b expression.

### TMEM39b regulates sorafenib-mediated inhibition of proliferation and migration in HCC cells

**Figure 5:**
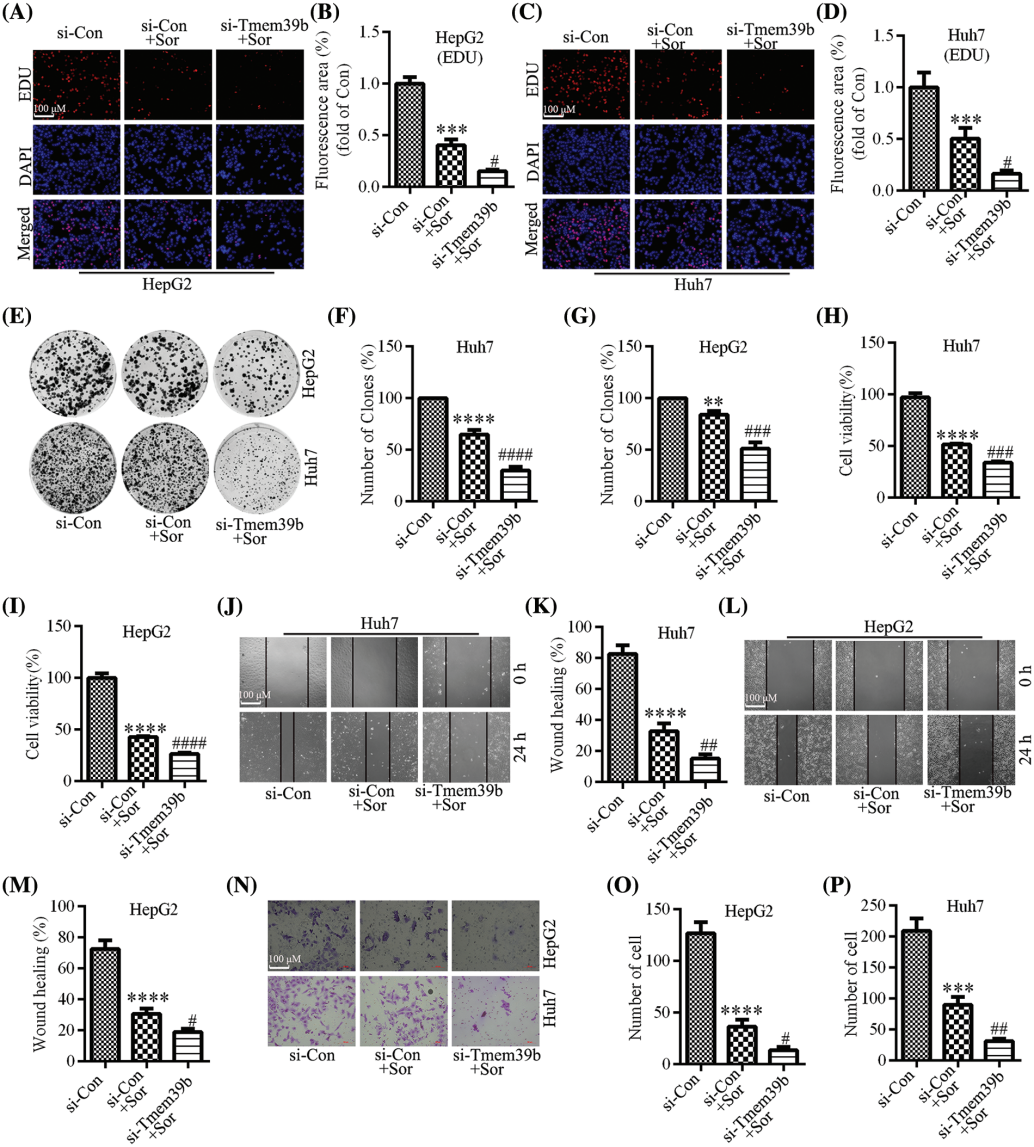
TMEM39b regulates sorafenib-mediated inhibition of proliferation and migration in HCC cells. (A–G) The cell proliferation ability of the si-Con group, si-Con + sorafenib group, and si-TMEM39b + sorafenib group. (H and I) The cell viability of si-Con group, si-Con + sorafenib group, and si-TMEM39b + sorafenib group. (J–P) The cell migration ability of the si-Con group, si-Con + sorafenib group, and si-TMEM39b + sorafenib group. ^#^*p* < 0.05, ^##^*p* < 0.01, ^###^*p* < 0.001, ^####^*p* < 0.0001 *vs*. Si-Con+Sor. **p < 0.01, ***p < 0.001, ****p < 0.0001 *vs*. Si-Con.

We identified through the EdU assay that the proliferative ability of HCC cells was significantly reduced after sorafenib treatment, with a more pronounced effect observed in the HCC cells from the si-TMEM39b group (Figs. 5A–5D). Similar results were observed in the colony formation assay (Figs. 5E–5G). Additionally, the CCK-8 assay confirmed a decline in HCC cell viability following sorafenib treatment, with the decrease more pronounced in the HCC cells of the si-TMEM39b group (Figs. 5H and 5I). Through the cell scratch assay, we observed a significant decrease in the migration ability of HCC cells following sorafenib treatment, and this decrease was more pronounced in the si-TMEM39b group (Figs. 5J–5M). Furthermore, consistent results were observed in the Transwell assay (Figs. 5N–5P). These results suggest that down-regulating the expression of TMEM39b can enhance the cytotoxic effect of sorafenib on HCC cells, resulting in decreased cell activity, proliferation, and migration.

### si-TMEM39b regulates sorafenib-induced ferroptosis in HCC cells

**Figure 6:**
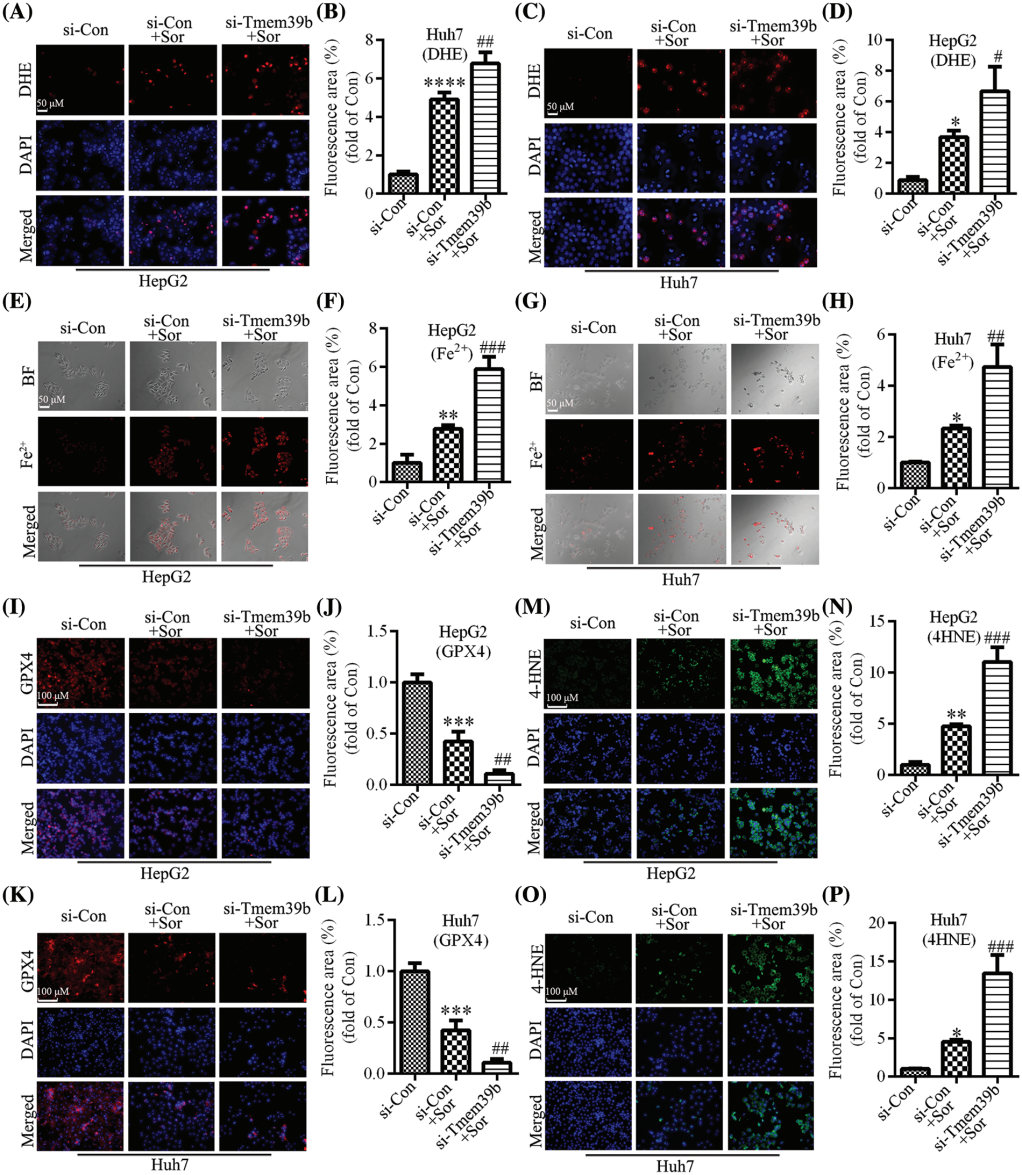
si-TMEM39b regulates sorafenib-induced ferroptosis in HCC cells. (A–D) The ROS content of the si-Con group, si-Con + sorafenib group, and si-TMEM39b + sorafenib group. (E–H) The Fe^2+^ content the si-Con group, si-Con + sorafenib group, and si-TMEM39b + sorafenib group. (I–L) The GPX4 content of the si-Con group, si-Con + sorafenib group, and si-TMEM39b + sorafenib group. (M–P) The 4-HNE content of the si-Con group, si-Con + sorafenib group, and si-TMEM39b + sorafenib group. ^#^*p* < 0.05, ^##^*p* < 0.01, ^###^*p* < 0.001, ^####^*p* < 0.0001 *vs*. Si-Con+Sor. *p < 0.05, **p < 0.01, ***p < 0.001, ****p < 0.0001 *vs*. Si-Con.

Using the DHE probe, we found that treatment with sorafenib induced an increase in ROS in HCC cells. Conversely, treating HCC cells with sorafenib followed by knockdown of TMEM39b expression resulted in a more pronounced increase in ROS (Figs. 6A–6D). Sorafenib significantly increased the levels of Fe^2+^ in HCC cells. In the si-TMEM39b group, the levels of Fe^2+^ in HCC cells treated with sorafenib showed a more significant increase (Figs. 6E–6H). Thus, reduced TMEM39b expression can promote the accumulation of Fe^2+^ in the cells. Similarly, sorafenib significantly decreased the expression of GPX4, and the expression of GPX4 in HCC cells of the si-TMEM39b + sorafenib group was even lower (Figs. 6I–6L). We found that treatment with sorafenib induced the increase of 4-HNE in HCC cells, while treatment with sorafenib based on TMEM39b knockdown increased the expression of 4-HNE more significantly (Figs. 6M–6P). Collectively, low expression of TMEM39b promoted sorafenib-induced ferroptosis. We hypothesize that TMEM39b may exert an inhibitory effect on sorafenib-induced ferroptosis in HCC cells.

## Discussion

Liver cancer ranks as the third leading cause of cancer-related mortality globally [1], and its incidence is expected to exceed 1 million by 2025 [20]. The prognosis of HCC is determined by tumor stage, with a 5-year survival rate exceeding 70% in early-stage HCC and a median survival of approximately 1–1.5 years in advanced-stage patients treated with systemic therapy [21]. Liver resection, ablation, and liver transplantation are potential treatments for liver cancer; however, they necessitate early diagnosis for favorable outcomes. Regrettably, early symptoms of liver cancer often remain latent, leading to diagnosis at an advanced stage for the majority of patients. Sorafenib has been the pioneering and consistently endorsed first-line therapy for advanced HCC patients [20,22]. Sorafenib, a multi-target tyrosine kinase inhibitor, primarily suppresses tumor cell proliferation and impedes tumor neovascularization through modulation of various intracellular and cell surface kinases. Sorafenib is widely used in patients with advanced liver cancer, yet susceptibility to drug resistance is prevalent in the population. In previous research efforts, significant resources were dedicated to studying the mechanism of drug resistance of sorafenib. Regrettably, these efforts appear to have fallen short in addressing the issue, prompting an exploration of potential new mechanisms that may contribute to sorafenib drug resistance in clinical practice. Notably, recent research has revealed an additional anti-tumor mechanism of sorafenib through the promotion of ferroptosis in tumor cells. Ferroptosis primarily arises from the imbalance between the generation and degradation of lipid ROS in the cell. The cystine glutamate transporter (system XC^-^) is a heterodimer consisting of solute carrier family 7 member 11 (SLC7A11) and solute carrier family 3 member 2. It has been demonstrated that inhibiting system XC^-^ impedes the absorption of glutathione, suppresses the reduction of GPXs activity, leads to the accumulation of lipid ROS, and consequently triggers ferroptosis in cells [23]. P53 has been identified as capable of repressing the expression of SLC7A11, blocking the absorption of glutathione, and inducing ferroptosis in cells [24,25]. The GPX family encompasses several members, with GPX4 currently recognized as the most pertinent to cell ferroptosis. GPX4 plays a role in enhancing cell tolerance to ferroptosis [26], while RSL3 can directly act on GPX4, impede its activity, and induce cell ferroptosis [27]. Furthermore, it has been observed that buthionine sulfoxime, benzhydryl-piperazine, and other cytokines exhibit similar functions to RSL3 and can inhibit GPX4 activity [28,29]. Recent studies have indicated that Yes-associated protein/transcriptional co-activator with PDZ-binding motif (YAP/TAZ) can regulate the expression of SLC7A11 [30]. In sorafenib-resistant HCC cells, YAP/TAZ is activated in the nucleus and binds to DNA fragments containing the transcriptional enhanced associate domain motif in the SLC7A11 gene promoter, thereby inducing the expression of SLC7A11, increasing the cellular level of glutathione, decreasing ROS levels, and inhibiting ferroptosis [30,31]. The P62 protein-kelch-like ECH-associated protein 1-nuclear factor erythroid 2-related factor serves as the core pathway for inhibiting ferroptosis. The up-regulation of downstream target genes of this pathway, such as metallothionein-1G [32] and ATP binding cassette subfamily C member 5 [33] can inhibit cell ferroptosis and enhance the resistance of HCC cells to sorafenib [34]. Moreover, miRNA plays a crucial role in the resistance of HCC cells to sorafenib. It has been demonstrated that miR-23a-3p targets the expression of acyl-CoA synthetase long-chain family member 4, consequently inhibiting ROS generation, alleviating ferroptosis in HCC, and enhancing HCC resistance to sorafenib [35]. With the completion of the Human Genome Project, a multitude of new genes have been identified, and their functions are currently under exploration. The TMEM protein family has been implicated in the development of tumors and immune-related diseases. TMEM39b, as a previously neglected gene, has rarely been studied. In the study, we transfected HCC cells with siRNA to down-regulate TMEM39b expression, resulting in weakened cell viability, proliferation, and invasion ability. Furthermore, the down-regulation of TMEM39b inhibited the RSL-3/GPX4 pathway and promoted ferroptosis in HCC cells induced by sorafenib. However, TMEM39b is highly expressed in ordinary HCC cells. In comparison, we observed that in ordinary HCC cells with high TMEM39b expression, the inhibition of the RSL-3/GPX4 pathway was not significant, and the accumulation of peroxide remained low even after sorafenib treatment. Common HCC cells did not exhibit evident ferroptosis; instead, they displayed high proliferative, invasive, and cellular activities.

Hence, we propose that TMEM39b can enhance the resistance of HCC cells to ferroptosis triggered by sorafenib via the RSL-3/GPX4 pathway. Additionally, this study is the first to unveil the potential significance of TMEM39b in the initiation and progression of HCC cells, thus offering novel insights for future treatment strategies.

## Data Availability

The data and materials supporting the current study are available from the corresponding author upon reasonable request.
